# Remote ischemic conditioning for the prevention of contrast-induced acute kidney injury in patients undergoing intravascular contrast administration: a meta-analysis and trial sequential analysis of 16 randomized controlled trials

**DOI:** 10.18632/oncotarget.18106

**Published:** 2017-05-23

**Authors:** Chang-Cheng Zhou, Wen-Tao Yao, Yu-Zheng Ge, Lu-Wei Xu, Ran Wu, Xiao-Fei Gao, Kai-Wei Song, Xiao-Min Jiang, Min Wang, Wen-Juan Huang, Yun-Peng Zhu, Liang-Peng Li, Liu-Hua Zhou, Zhong-Le Xu, Sheng-Li Zhang, Jia-Geng Zhu, Wen-Cheng Li, Rui-Peng Jia

**Affiliations:** ^1^ Department of Urology, Nanjing First Hospital, Nanjing Medical University, Nanjing, China; ^2^ Department of Cardiology, Nanjing First Hospital, Nanjing Medical University, Nanjing, China; ^3^ Department of Nephrology, Nanjing First Hospital, Nanjing Medical University, Nanjing, China; ^4^ Department of Cardiothoracic Surgery, Nanjing First Hospital, Nanjing Medical University, Nanjing, China; ^5^ Department of Radiology, Nanjing First Hospital, Nanjing Medical University, Nanjing, China

**Keywords:** remote ischemic conditioning, contrast-induced acute kidney injury, randomized controlled trial, meta-analysis, trial sequential analysis

## Abstract

**Objective:**

We conducted this meta-analysis to examine the effect of remote ischemic conditioning (RIC) on contrast-induced acute kidney injury (CI-AKI) in patients undergoing intravascular contrast administrationon.

**Methods:**

Pubmed, Embase, and Cochrane Library were comprehensively searched to identify all eligible studies by 15th March, 2017. Risk ratio (RR) and weighted mean difference with the corresponding 95% confidence intervals (CI) were used to examine the treatment effect. The heterogeneity and statistical significance were assessed with Q-test and Z-test, respectively.

**Results:**

A total of 16 RCTs including 2175 patients were eventually analyzed. Compared with the control group, RIC could significantly decrease the incidence of CI-AKI (RR=0.58; 95% CI: 0.46, 0.74; *P* < 0.001), which was further confirmed by the trial sequential analysis. Subgroup analyses showed that remote ischemic preconditioning (RIPrC) and remote ischemic postconditioning (RIPoC) were both obviously effective, and perioperative hydration might enhance the efficiency of RIC. RIC also significantly reduced the major adverse cardiovascular events within six months.

**Conclusion:**

RIC, whether RIPrC or RIPoC, could effectively exert renoprotective role in intravascular contrast administration and reduce the incidence of relevant adverse events.

## INTRODUCTION

Contrast-induced acute kidney injury (CI-AKI) has become the third major cause of hospital-acquired renal insufficiency due to the widespread application of contrast medium in the clinical setting, which is associated with increased morbidity and mortality, prolonged hospital stay, and aggravated economic burden [1, 2]. Low osmolar contrast medium, hydration protocols, and prophylactic utility of drugs have been introduced to prevent CI-AKI [3-5]; however, the incidence of CI-AKI remains significant [3, 6]. Furthermore, undetermined adverse effects of prophylactic drugs and limited utilization of hydration deliver an insufficient role in renal protection [7, 8]. Therefore, a safe, feasible, and effective strategy is urgently warranted to prevent CI-AKI.

Remote ischemic conditioning (RIC) is a non-pharmacological approach induced by several cycles of transient nonlethal ischemia and reperfusion to one remote organ or issue, which could protect another organ or tissue from prolonged lethal ischemia reperfusion injury (IRI) [9, 10]. According to diverse inducing time points, RIC is commonly categorized into three types: remote ischemic preconditioning (RIPrC), remote ischemic perconditioning (RIPeC), and remote ischemic postconditioning (RIPoC) [11-13]. RIC was initially performed to attenuate IRI of the heart [14], and rapidly extended to other vital organs including kidney, brain, intestine, and lung [15-18]. Although the exact mechanisms of RIC remain ambiguous, RIC has been proven to exert nephronprotective function in patients undergoing renal or non-renal surgery [17-20].

CI-AKI is a multifactorial pathophysiological condition with two major contributors, namely, direct toxicity and renal IRI caused by contrast medium [21-24]. Encouraged by the positive results in animal studies [25, 26], various clinical studies have been conducted to explore the potential impacts of RIC on CI-AKI in patients intravascularly administrated with contrast medium [27]. However, inconclusive results were obtained because of the limited sample size and various study protocols. Previous meta-analysis studies were focused on the role of RIC in patients undergoing percutaneous coronary interventions (PCI) or coronary angiography (CA) [28, 29]. Hence, we conducted this meta-analysis to evaluate the clinical safety and efficacy of RIC by pooling data from all eligible trials about intravascular contrast administration for diagnostic or therapeutic aims [7, 8, 22, 30-42].

## RESULTS

### Search results and study characteristics

**Figure 1:**
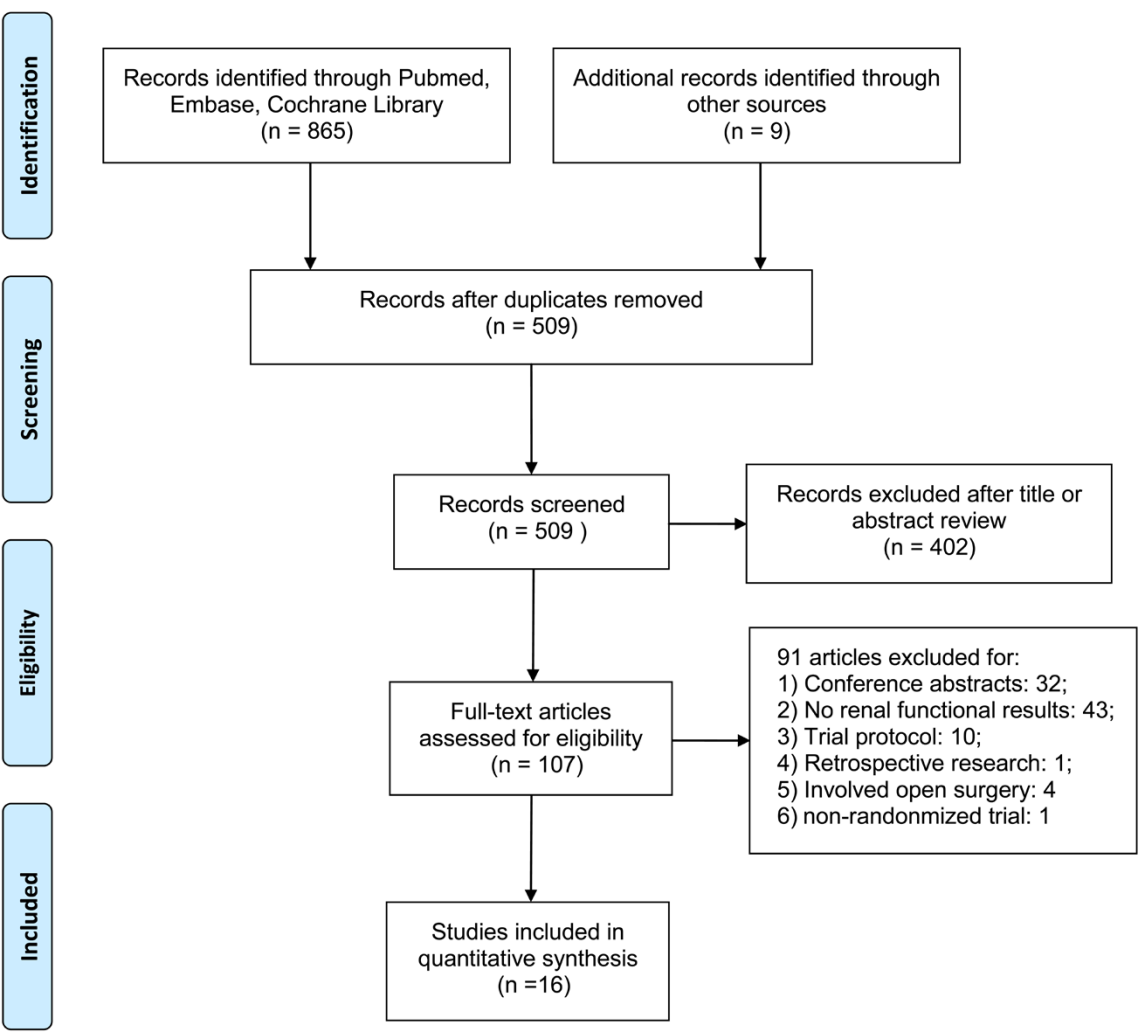
Flow diagram of study selection Description: a total of 16 studies were included in this meta-analysis after a comprehensive study selection.

A total of 874 citations were generated via the search strategy, of which 365 duplicates and 402 clearly irrelevant studies were excluded after reading the title and abstract. After full-text assessment of the remaining 107 potentially relevant studies, 91 were removed for: trial protocol, conference abstract, no renal functional results, retrospective observational cohort design, or study subjects involved open surgery. Finally, a total of 16 articles were eligible for this study [7, 8, 22, 30-42], and the detailed screening process was presented in Figure 1.

A total of 2175 patients were enrolled in the 16 randomized controlled trials (RCTs), where 1093 participants were randomly assigned to the RIC group and 1082 to the control group. In these trials, contrast medium was utilized for PCI, CA, enhanced computed tomography scan, endovascular aneurysm repair, and other operation for diagnostic or therapeutic purpose. The detailed characteristics of included studies were shown in Tables 1, Supplementary Table S1 in Supplementary and Table 2.

**Table 1:** Characteristics of included trials

Author	Year	No. of patients	Mean age	Males (%)	Operation name	Contrast Dose (mL)	Hydration
Kahlert	2017	50/50	80.4±6.4/83.1±4.9	44/50	TAVI	183.6±68.0/201.4±71.0	B and A
Balbir	2016	51/51	67.8±7.6/69.0±8.6	45/49	PCI	197.5±114.3/196.4±118.8	B and A
Yamanaka	2015	47/47	67±12/67±15	76/76	PCI	177±53/199±87	B and A
Menting	2015	36/36	71±11/73±8.5	39/58	Diagnostic/treatment	99±29/98±29	B and A
Healy	2015	43/44	63±8.9/62±7.4	51/59	Enhanced CT scan	90 to 120	B
Gholoobi	2015	25/26	67.1±12.5/70.3±11.2	72/46	CA/PCI	77.7	B and A
Xu	2014	102/98	69.1±3.8/68.9±2.9	66/70	DES implantation	171.8±37.9/163.3±39	NO
Savaj	2014	48/48	63.0±8.9/60.9±9.6	35/29	CA	126.6±77.2/123.8±66.6	B
Lavi 1	2014	120/120	63.6±10.3/63.7±9.7	65/70	PCI	190±97/185±87	NO
Lavi 2	2014	120/120	64.9±9.6/63.7±9.7	68/70	PCI	190±84/185±87	NO
Crimi	2014	47/48	61±11/56±11	41/43	PCI	211±55/229±72	NO
Luo	2013	101/104	59.2±10.3/59.3±9.5	78/78	DES implantation	154±46/145±41	B and A
Igarashi	2013	30/30	71.3±8.1/70.8±7.6	20/23	CA	92.9±33.2/91.8±39.4	B and A
Deftereos	2013	113/112	68±7.4/68±4.4	65/62	PCI	270±59.3/265±37	B and A
Er	2012	50/50	73.2±9.1/72.7±11.4	68/74	CA+PCI	124±44/103±41	B and A
Walsh	2009	18/22	74±6.7/76±10.4	100/100	EVAR	309±137/286±93	NO
Hoole	2009	104/98	63.2±10.1/61.8±10.3	84/74	PCI	196.7±80.1/187.5±74.2	NO

PCI: percutaneous coronary intervention; CT: computed tomography; DES: drug-eluting stent; CA: coronary angiography; EVAR: endovascular aneurysm repair; TAVI: transcatheter aortic valve implantation; B: hydration performed before contrast administration; A: hydration performed after contrast administration.

**Table 2:** Detailed information of operation process

Author	Year	RIC type	RIC protocol	Conditioning organ	CI-AKI Definition
Kahlert	2017	preconditioning	3×5/5 min	arm	50% rise or 0.3 mg/dL increase of Scr within 72h
Balbir	2016	preconditioning	3×5/5 min	arm	increase of Scr ≥0.5 mg/dL or ≥25% above baseline with 48h
Yamanaka	2015	preconditioning	3×5/5 min	arm	increase of Scr >0.5 mg/dL or >25% above baseline within 72h
Menting	2015	preconditioning	4×5/5 min	arm	increase of Scr >0.5 mg/dL or >25% above baseline within 72h
Healy	2015	preconditioning	4×5/5 min	arm	increased SCr with eGFR <90ml/min/1.73m2 within 48h
Gholoobi	2015	preconditioning	4×5/5 min	arm	increase of Scr >0.3 mg/dL above baseline within 48h
Xu	2014	preconditioning	3×5/5 min	arm	increase of Scr>25% above baseline within 16h
Savaj	2014	preconditioning	3×5/5 min	arm	30% rise or 0.3 mg/dL increase of Scr within 24h
Lavi 1	2014	postconditioning	3×5/5 min	arm	increase of Scr >44 μmol/L or >25% above baseline within 24h
Lavi 2	2014	postconditioning	3×5/5 min	thigh	increase of Scr >44 μmol/L or >25% above baseline within 24h
Crimi	2014	postconditioning	3×5/5 min	thigh	increase of SCr ≥25% above baseline within 24h
Luo	2013	preconditioning	3×5/5 min	arm	increase of Scr >44.2 μmol/L or >25% above baseline within 16h
Igarashi	2013	preconditioning	4×5/5 min	arm	increase of L-FABP >17.4µg/g Cr or >25% above baseline within 24h
Deftereos	2013	postconditioning	4×30/30 sec	heart	increase of Scr >0.5 mg/dL or >25% above baseline within 96h
Er	2012	preconditioning	4×5/5 min	arm	increase of Scr ≥0.5 mg/dL or ≥25% above baseline with 48h
Walsh	2009	preconditioning	2×10/10 sec	thigh	decrease of eGFR ≥20% above baseline within 24h
Hoole	2009	preconditioning	3×5/5 min	arm	increase of Scr>25% above baseline within 24h

RIC: remote ischemic conditioning; CI-AKI: contrast-induced acute kidney injury; SCr: serum creatinine; eGFR: estimated glomerular filtration rate; L-FABP: liver-type fatty acidbinding protein.

### Risk of bias

**Figure 2:**
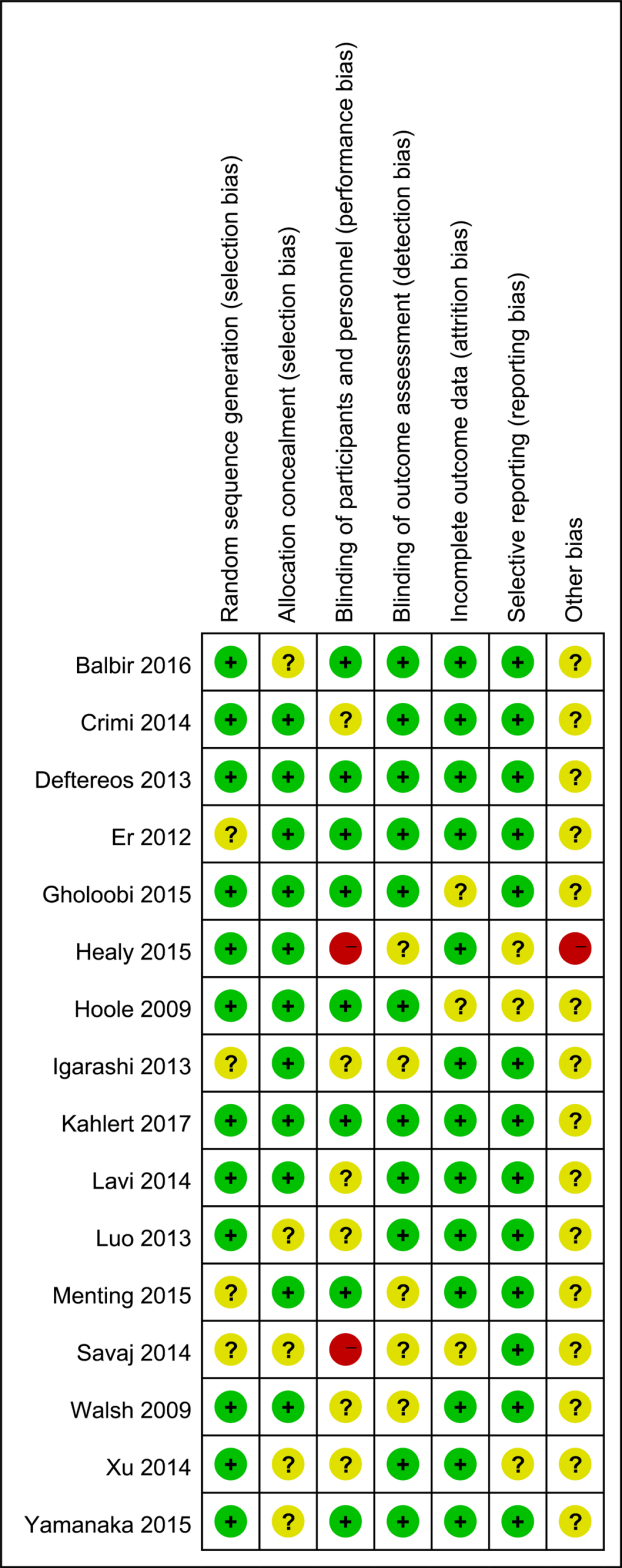
Risk of bias summary of all included randomised clinical trials Green+: low risk; Red-: high risk; Yellow?: unclear risk.

The quality of the 16 trials was assessed independently by two authors (Xiao-Fei Gao and Ran Wu) using the Cochrane Collaboration tool [43]. Two trials scored high risk of bias for the absence of appropriate blinding [7, 32] and non-strict control [32]. The detailed quality assessments were shown in Figure 2.

### Study outcomes

#### Incidence of CI-AKI

**Figure 3:**
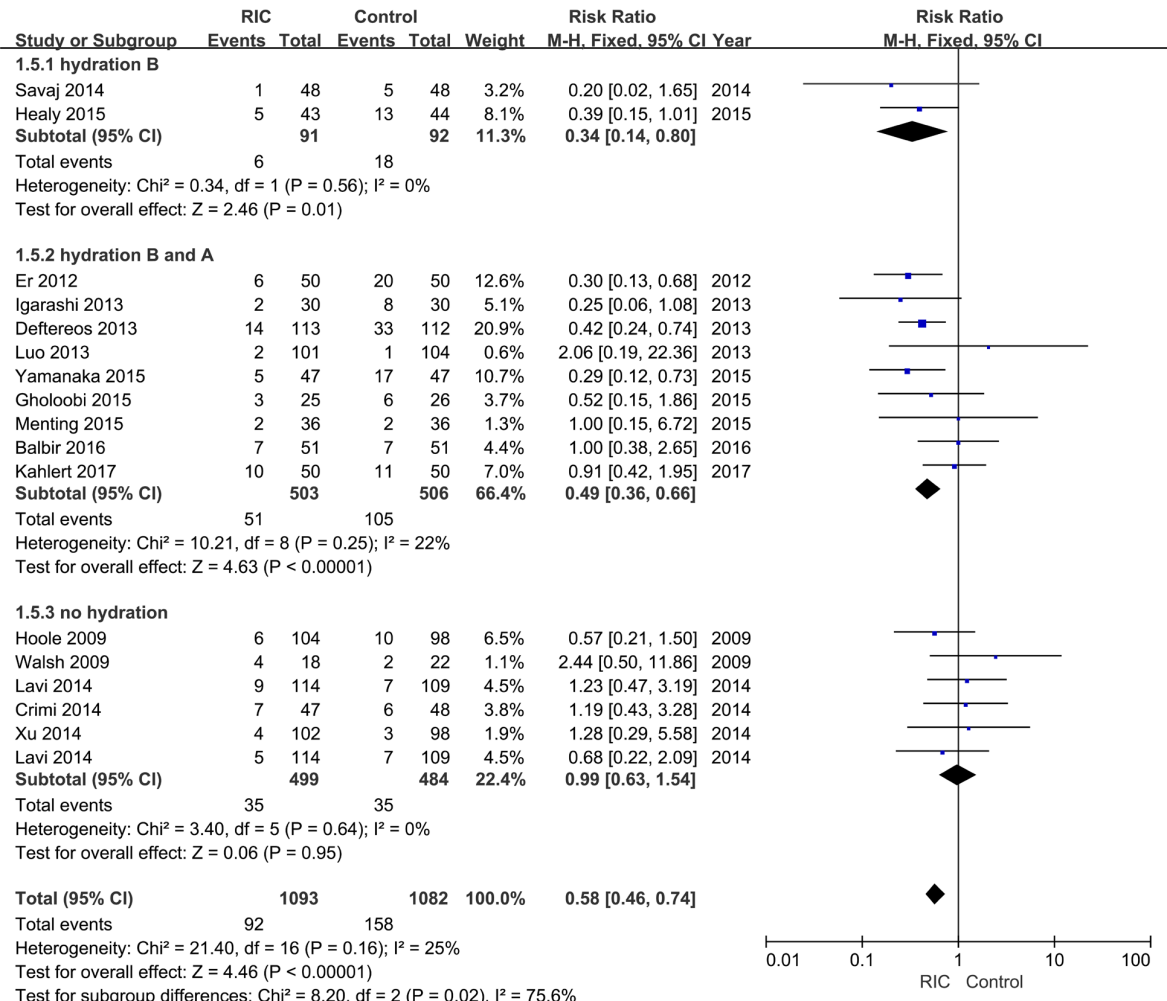
Forest plot with 95% confidence interval in CI-AKI incidence Studies are sorted by performance of hydration during perioperative period.

**Figure 4:**
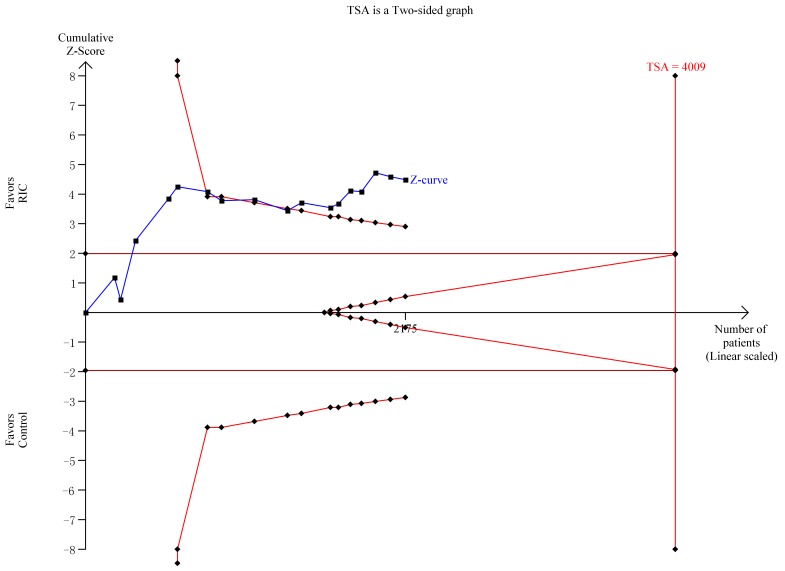
Trial sequential analysis of the CI-AKI incidence As shown in panel, the cumulative z curve for rates of CI-AKI did cross the sequential monitoring boundaries, however the required optimal sample size was not achieved.

All 16 RCTs included in this study reported the incidence of CI-AKI. A significant decline in CI-AKI incidence cloud be observed in the RIC group compared with the control group based on the fixed effect mode (RR = 0.58; 95% CI: 0.46, 0.74; *P* < 0.001; Figure 3). Furthermore, the trial sequential analysis (TSA) revealed that the cumulative z curves for the incidence of CI-AKI crossed the sequential monitoring boundaries (Figure 4), which indicated that RIC had a protective effect on CI-AKI compared with the control with 25% relative risk reduction (RRR), and the trend was unlikely to be altered by future RCTs.

#### Subgroup analyses

Hydration was administrated in eleven trials. Two RCTs conducted hydration only before contrast administration, while the other nine trials applied it before and after contrast administration. Subgroup analysis demonstrated that RIC could statistically reduce the risk of CI-AKI in patients undergoing perioperative hydration, whether it was conducted only before contrast infusion (RR = 0.34; 95% CI: 0.14, 0.80; *P* = 0.01; Figure 3) or combined with post-operation (RR = 0.49; 95% CI: 0.36, 0.66; *P* < 0.001; Figure 3). However, absence of hydration rendered the RIC inefficient (RR = 0.99; 95% CI: 0.63, 1.54; *P* = 0.95; Figure 3).

Mean contrast dose varied between studies, and the included studies were categorized into three groups: low level ( < 100 ml, four RCTs), medium level (100∼200 ml, nine RCTs), and high level (>200 ml, three RCTs) [41]. RIC effectively reduced the incidence of CI-AKI in the low (RR = 0.42; 95% CI: 0.23, 0.79; *P* = 0.007) and medium level (RR = 0.61; 95% CI: 0.45, 0.84; *P* = 0.002). However, the renoprotective effects of RIC become unconspicuous in the high level (RR = 0.89; 95% CI: 0.32, 2.47; *P* = 0.83).

Different types of RIC were conducted in these included studies. Among these studies, thirteen trials used RIPrC, while the remaining three performed RIPoC. Both RIPrC and RIPoC could significantly prevent CI-AKI (RIPC: RR = 0.55; 95% CI: 0.41, 0.74; *P* < 0.001; RIPoC: RR = 0.65; 95% CI: 0.44, 0.97; *P* = 0.03).

Diverse protocols were applied during the RIC procedures. Nine studies conducted conditioning protocol A (CPA, three cycles of 5 min of ischemia and 5 min of reperfusion) to induce RIC, five studies conducted conditioning protocol B (CPB, four cycles of 5 min of ischemia and 5 min of reperfusion), and the remaining two studies utilized other different protocols (Table 2). RIC implemented through CPB obviously reduced CI-AKI incidence (RR = 0.37; 95% CI: 0.23, 0.61; *P* < 0.001), while in CPA group, the reduction did not reach the significant level (RR = 0.74; 95% CI: 0.54, 1.03; *P* = 0.08).

Additionally, twelve trials utilized upper limb as a conditioning organ, two trials used lower limb, one trial included three groups (upper limb, lower limb and control group), and the remaining one trial applied myocardium. Subgroup analysis indicated that RIC induced by upper limb showed a significant decline in CI-AKI incidence (RR = 0.52; 95% CI: 0.39, 0.70; *P* < 0.001). However, no significant protective effect of RIC induced by low limb was observed (RR = 1.36; 95% CI: 0.72, 2.56; *P* = 0.34).

Moreover, multiple definitions of CI-AKI were adopted in the included RCTs. Seven trials applied traditional definition A (TDA, increase in serum creatinine [SCr]>0.5 mg/dl or >25%), three trials used traditional definition B (TDB, SCr> 25% increase), and the remaining six trials used other self-defined definitions. According to TDA, RIC showed a significant role in protecting CI-AKI from contrast damage (RR = 0.53; 95% CI: 0.38, 0.72; *P* < 0.001), as well as the other self-defined definitions (RR = 0.57; 95% CI: 0.37, 0.90; *P* = 0.01). By contrast, TDB was insufficient in identifying CI-AKI occurrence (RR = 0.87; 95% CI: 0.47, 1.63; *P* = 0.67). All detailed results of subgroup analyses were summarized in Table 3.

**Table 3:** Subgroup analysis of CI-AKI incidence

Category	No. of trials	RR	95% CI	*P*	*P*_heterogeneity_
Total	16	0.58	0.46, 0.74	**<0.001**	0.16
Hydration					
Before	2	0.34	0.14, 0.80	**0.01**	0.56
Before and after	9	0.49	0.36, 0.66	**<0.001**	0.25
NO	5	0.99	0.63, 1.54	0.95	0.64
Mean contrast dose					
low	4	0.42	0.23, 0.79	**0.007**	0.70
medium	9	0.61	0.45, 0.84	**0.002**	0.18
high	3	0.89	0.32, 2.47	0.83	**0.04**
RIC type					
preconditioning	13	0.55	0.41, 0.74	**<0.001**	0.20
postconditioning	3	0.65	0.44, 0.97	**0.03**	0.15
RIC protocol					
CPA	9	0.74	0.54, 1.03	0.08	0.39
CPB	5	0.37	0.23, 0.61	**<0.001**	0.76
Other	2	0.86	0.16, 4.71	0.87	**0.04**
Conditioning organ					
arm	13	0.52	0.39, 0.70	**<0.001**	0.41
thigh	3	1.36	0.72, 2.56	0.34	0.73
heart	1	0.42	0.24, 0.74	**0.003**	–
CI-AKI definition					
TDA	7	0.53	0.38, 0.72	**<0.001**	0.16
TDB	3	0.87	0.47, 1.63	0.67	0.50
Self-defined	6	0.57	0.37, 0.90	**0.01**	0.19

CI-AKI: contrast-induced acute kidney injury; RIC: remote ischemic conditioning; CPA: conditioning protocol A; CPB: conditioning protocol B; TDA: traditional definition A; TDB: traditional definition B. Statistically significant results are shown in bold.

#### Meta-regression analysis

The results of meta-regression indicated that there were no significant correlation between the renoprotective role of RIC and potential confounders such as percentage of prior other disease (including diabetes, hypertension, dyslipidaemia and coronary artery disease), previous contrast administration, baseline SCr, duration of RIC, and contrast dose. However, RIC tended to enhance its renoprotection with a marginal statistical significance along with a perioperative hydration (*P* = 0.05) (Supplementary Figure S1).

#### Postoperative kidney biomarkers

**Figure 5:**
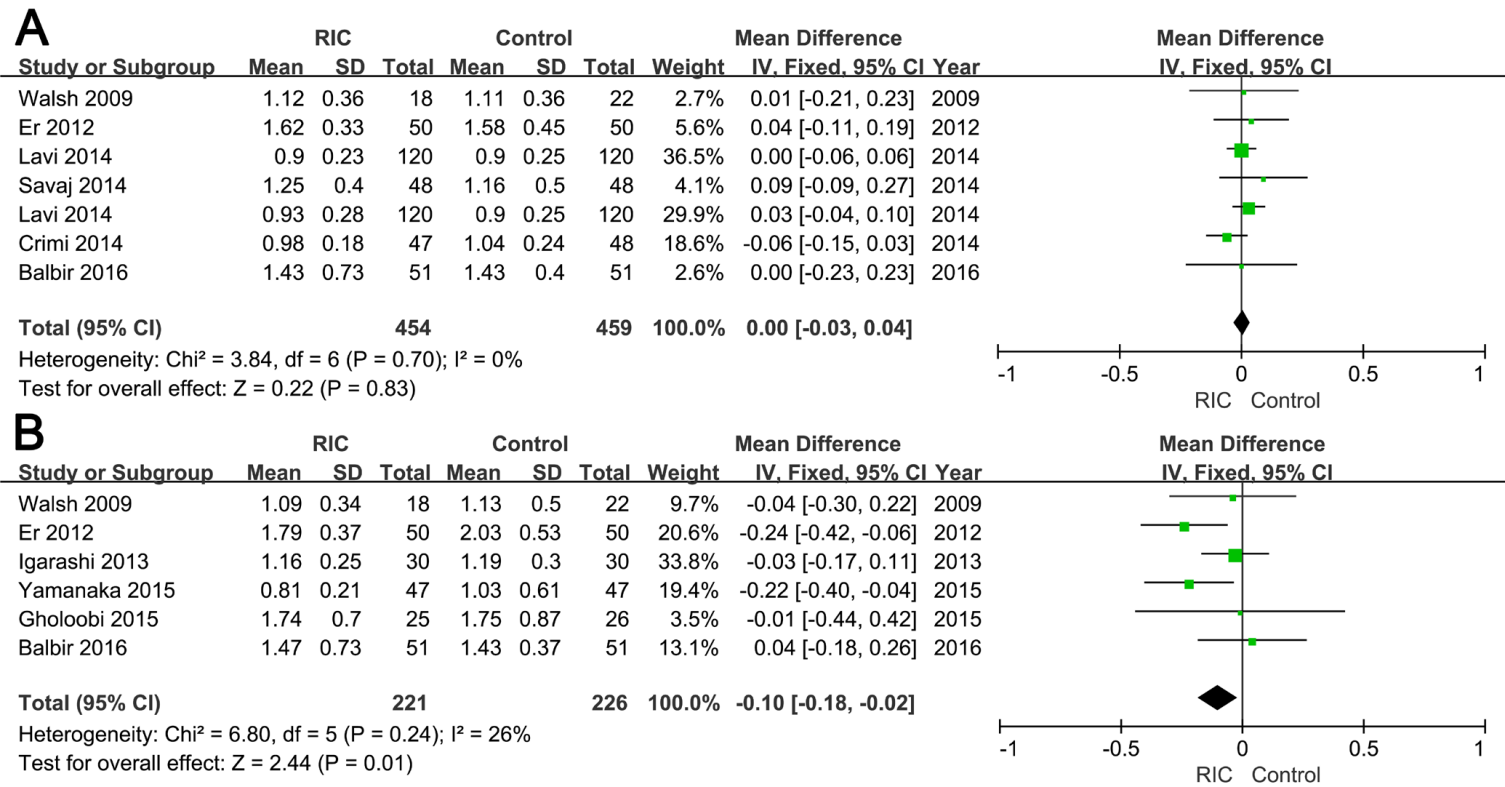
Forest plot with 95% confidence interval in postoperative 24h (A) and 48h (B) serum creatinine in patients treated with RIC compared with controls

Data regarding SCr was assessed in nine trials. As presented in Figure 5, a significant difference could be detected in SCr levels at 48 h (WMD = -0.10; 95% CI: -0.18, -0.02; *P* = 0.01; Figure 5B), even though SCr at 24 h postoperative did not differ significantly between two groups (WMD = 0; 95% CI: -0.03, 0.04; *P* = 0.83; Figure 5A).

#### Mortality and major adverse cardiovascular events (MACEs)

**Figure 6:**
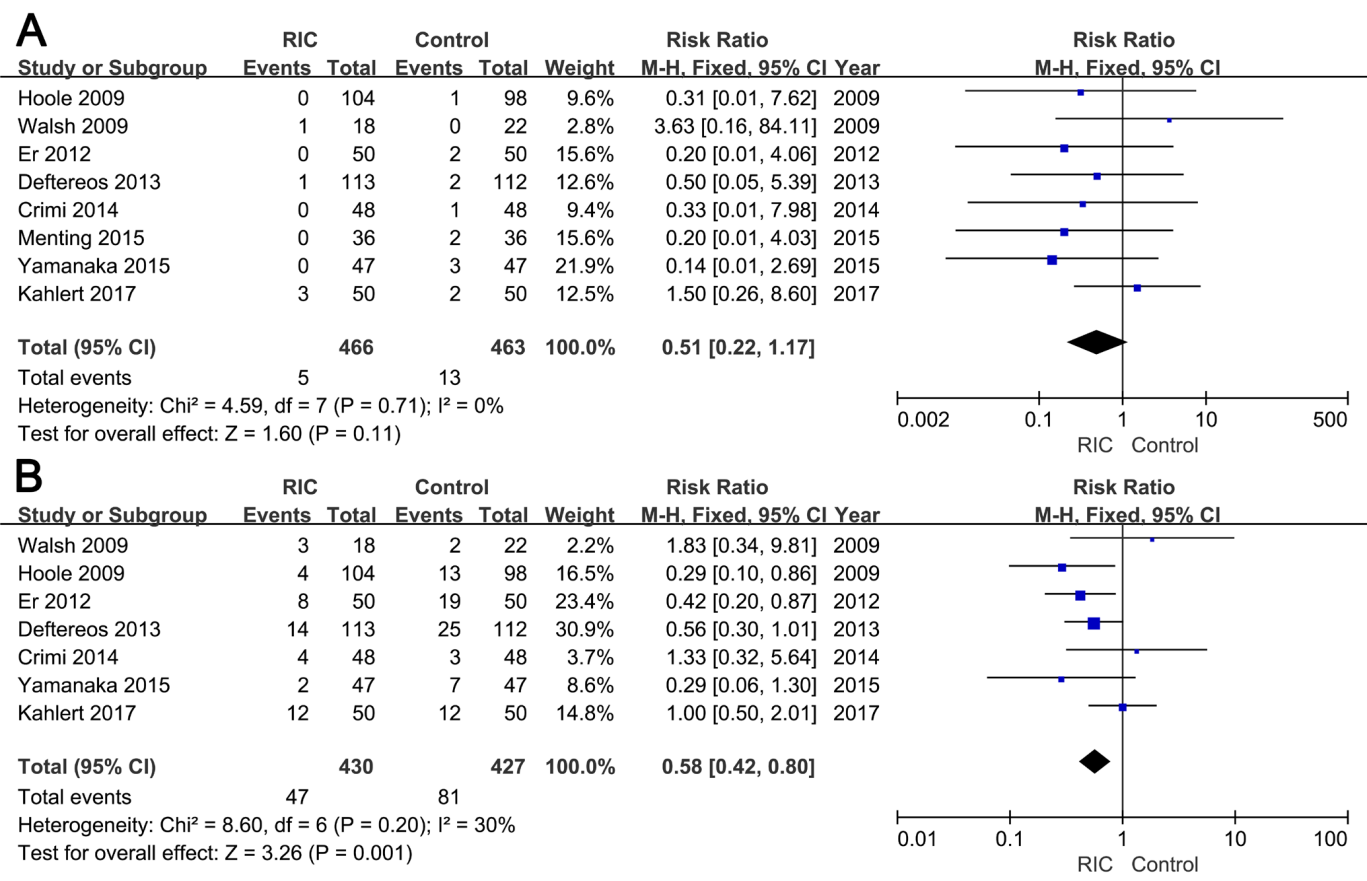
Forest plot with 95% confidence interval in incidence of mortality (A) and major adverse cardiovascular events (B) in patients treated with RIC compared with controls

All-cause mortality was mentioned in eight trials. A trend of decline in the mortality within six months could be observed s between two groups, however, the decrease did not reach statistical significance (RR = 0.51; 95% CI: 0.22, 1.17; *P* = 0.11; Figure 6A). Seven trials reported MACEs during the six-month follow-up, and the incidence of MACEs was significantly decreased in the RIC group (RR = 0.58; 95% CI: 0.42, 0.80; *P* = 0.001; Figure 6B).

### Heterogeneity evaluation and sensitivity analysis

No significant heterogeneity was observed in the global analyses, except in the subgroup analysis of CI-AKI in terms of contrast dose and conditioning protocols, where the random effects model was applied. These heterogeneities might be attributed to the relatively large difference of sample size between trials included in the two subgroups. Moreover, sensitivity analyses were conducted on all outcomes to detect the potential role of each individual study on the pooled results. The results revealed that no single study delivered substantial power to alter the pooled outcomes significantly, except one study in the subgroup of high contrast dose regarding the primary outcome [38].

### Publication bias and quality assessment

Both Begg’s funnel plot and Egger’s linear regression test were performed to assess the publication bias of the currently available literature. The funnel plots did not reveal any apparent asymmetry (Supplementary Figure S2 and Supplementary Table S2). Egger’s test also did not reveal any evidence of publication bias (Supplementary Table S2). Furthermore, the overall quality of evidence for each endpoints was rated as moderate by Grading of Recommendations Assessment, Development and Evaluation (GRADE) instrument for the presence of risk of bias, inconsistency, indirectness, or imprecision (Supplementary Table S3).

## DISCUSSION

CI-AKI is a frequent and severe complication in patients intravascularly administrated with contrast medium [39], signifying the need for more protective approaches. To the best of our knowledge, this report is the first meta-analysis to comprehensively evaluate the efficacy and safety of RIC in intravascular contrast administration. The results demonstrated that RIC could significantly prevent CI-AKI, which was further supported by TSA.

Although the precise underlying mechanisms remain ambiguous, mounting evidence has proved renal IRI as a major contributor of CI-AKI [21, 44]. RIC has been demonstrated as a potent approach to prevent renal IRI both experimentally and clinically [20]. Hoole and colleagues initially reported in 2009 that RIC could prevent CI-AKI in PCI [22]. Subsequently, numerous clinical trials have been conducted in patients administrated with contrast medium [7, 8, 30-40]. However, due to the different study designs and limited sample size, inconclusive results were obtained. In the current study, we integrated the data by pooling all eligible studies, and found that RIC could significantly prevent CI-AKI, in the terms of both primary and secondary endpoints, and the robustness of evidence for each outcomes was moderate. Moreover, the subgroup analyses indicated that: 1) hydration, conducted either before or after contrast administration, may positively promote RIC to prevent CI-AKI; 2) RIC was effective at low and medium doses of contrast administration; 3) Both RIPrC and RIPoC were functional; 4) RIC induced through CPB, rather than other conditioning protocols, revealed a significant renoprotective role; 5) Using upper limb as the remote conditioning organ could obviously reduce the risk of CI-AKI; 6) TDB might be insufficient in identifying CI-AKI occurrence.

Among all available strategies for preventing kidneys from contrast damage, sufficient intravenous hydration (commonly induced by infusion of isotonic saline) is one of the most beneficial method [24]. Our findings showed that hydration, conducted before or after contrast infusion, could enhance the efficiency of RIC compared with trials without hydration, and the two renoprotective strategies might have synergism in the protection of contrast damage. However, hydration was frequently limited in numerous patients with subclinical heart and kidney dysfunction [7]. Therefore, given the synergistic effect and limitation, hydration should be performed as sufficiently and safely as possible when conducting RIC during contrast administration.

The administrated contrast dose is significantly associated with the incidence of CI-AKI [24, 45], which could influence the renoprotective effects of RIC. Savaj et al. [7] performed a stratified analysis concerning diverse risk factors to compare the change of SCr in groups. The results indicated that both RIC-treated and control groups revealed a remarkable change of SCr when the contrast dose >60 ml. Subgroup analysis of the present study also further demonstrated that RIC was significantly effective in studies involving low and medium dose contrast rather than in high doses. Unfortunately, the results of meta-regression did not yield a significant dose-effect, and more well-designed RCTs are warranted to further confirm this conclusion.

Additionally, we explored the influence of RIC executing process in different aspects: 1) conditioning type. The initial conditioning type was RIPrC, and RIPoC was regarded as a further evolution for the limitation of RIPrC in urgent situations [34]. Accordingly, our findings showed that both RIPrC and RIPoC could significantly decrease the incidence of CI-AKI. Moreover, Ovize et al. [46] proposed that postconditioning should be performed initially at the time of reperfusion instead of a delay to achieve a protective effect. 2) conditioning protocol. RIC is generally induced by three or four cycles of 5 min of ischemia and reperfusion. Subgroup analysis revealed that CPB (four cycles) was significantly effective in renal protection, while CPA (three cycles) was not. In accordance with our inference, pre-clinical trials induced by Dong et al. [47] and Lu et al. [48] confirmed the relevance between conditioning cycles and its efficiency, and demonstrated that more cycles rendered the RIC more powerful. 3) conditioning organ. Arm and thigh are widely treated as conditioning organs to induce RIC because of their security and convenience [30]. Previous evidence has indicated that a large organ combined with simultaneous interventions was more effective compared with smaller organ and single intervention in either animal models or human beings [49, 50]. However, our findings delivered contradictory outcomes, in which conditioning with the arm significantly reduced the risk of CI-AKI rather than the thigh, which should be validated in future RCTs.

The various definitions of CI-AKI can directly and markedly influence the incidence of CI-AKI. Trials included in the present study utilized the change in SCr, estimated glomerular filtration rate, and even urinary liver-type fatty acid binding protein (L-FABP) to evaluate the degree of kidney injury. Subgroup analysis based on varied definitions uncovered an inadequacy of the TDB which would be biased and miss the cases with relatively high baseline level. Moreover, TDA based on SCr and definitions based on L-FABP were performed together by Igarashi et al. [37] to evaluate the incidence of CI-AKI. The results showed that no significant difference according to TDA, whereas remarkable distinction was observed on L-FABP.

We also measured the SCr level at 24 and 48 h postoperative to assess renal injury. Pooled results indicated that SCr at 48 h postoperative obviously differed between the two groups, but not at 24 h postoperative. In accordance with our findings, Luo et al. [36] demonstrated no significant difference when CI-AKI was evaluated at 16 h postoperative. Moreover, they held that most of contrast-induced nephropathy should have been induced at 48 h postoperative rather than at an earlier time. Although SCr is widely used, it is treated as a suboptimal biomarker because it cannot rapidly reflect the degree of kidney injury [37, 51]. Thus, growing trials utilize more sensitive biomarkers, such as L-FABP, cystatin C, and neutrophil gelatinase-associated lipocalin, to evaluate renal function after contrast administration [37, 39].

We introduced mortality and MACEs within six months to assess the security of RIC. In accordance with our hypothesis, RIC was proven to reduce the CI-AKI rates without aggravating the incidence of adverse events.

Several limitations of this study should be acknowledged when interpreting the results. First, the duration of measuring relevant biomarkers to define CI-AKI differed among studies, from 16 h to 96 h, which could directly influence the rates of CI-AKI. Second, SCr utilized in most of the included trials was not sensitive to detect kidney injury after contrast administration, resulting in a relatively low incidence of CI-AKI. Third, renal function was evaluated just by some short-term outcomes without a long-term follow-up. Fourth, most of the trials included in the present analysis were associated with PCI or CA, only four RCTs referred to other contrast-related operations. Finally, only three double-blind trials were included in our study, the outcomes would be interfered by aware participants.

In summary, our work indicated that RIC, either RIPrC or RIPoC, could effectively exert renoprotective role in diagnostic or therapeutic intravascular contrast administration and reduce the incidence of relevant adverse events. Moreover, well-designed RCTs with unified criteria and large sample size are needed to evaluate the exact efficacy and safety of RIC in contrast administration.

## MATERIALS AND METHODS

### Identification of eligible studies

This meta-analysis was performed and presented in accordance with the guidelines of the Preferred Reporting Items for Systematic reviews and Meta-Analyses [52]. We conducted a comprehensive electronic search of Pubmed, Embase and Cochrane Library (last update: 15th March, 2017) to identify all eligible studies. The following keywords were used in multiple combinations: remote ischemic preconditioning, remote ischemic postconditioning, remote ischemic conditioning, or remote ischemic perconditioning; randomized controlled trial or controlled clinical trial or randomized trial. We also searched ClinicalTrials.gov website, Google Scholar and Open Grey for other potential eligible RCTs. Furthermore, the reference lists of reviews and retrieved articles were manually searched for additional records. No restriction was executed during the literature search.

### Selection criteria

Studies were assessed independently by two investigators (Chang-Cheng Zhou, and Yu-Zheng Ge) according to the following predesigned inclusion criteria: (1) study design as prospective RCTs; (2) patients received a recorded dose of intravascular contrast administration; (3) treatment group was administrated with one type of RIC (RIPrC, RIPeC, or RIPoC); and (4) sufficient data were provided to evaluate the short- or long-term outcomes. The trial protocols, conference abstracts, retrospective researches, and studies about open surgery were discarded. Studies without detailed information were also excluded, after the efforts to extract data from the original paper or contact the corresponding authors failed.

### Data extraction

Two authors (Chang-Cheng Zhou and Wen-Tao Yao) gathered data from all eligible studies independently using a predesigned data collection form. The following information was extracted: primary outcome, incidence of CI-AKI; secondary outcomes, SCr at 24 h and 48 h post administration, mortality and MACEs in six months. Concurrently, the following data were also extracted: last name of first author, publication year, demographic characteristics of the patients, type of operation, RIC type and protocol, definition of CI-AKI, and administration of hydration. Discrepancies were resolved via discussion with another two investigators (Yu-Zheng Ge and Rui-Peng Jia).

### Assessment of risk of bias

The Cochrane Collaboration tool was used to assess the methodological quality of each included studies [43], which including the following domains: random sequence generation, allocation concealment, blinding of patients, personnel and outcome assessment, incomplete outcome data, selective reporting, and other sources of bias. Each item was judged as “low”, “unclear”, or “high” risk of bias.

### Statistical analysis

Weighted mean difference (WMD) and the 95% confidence interval (CI) were calculated for continuous variables, while risk ratio (RR) with its corresponding 95% CI was yielded for dichotomous variables. We conducted subgroup analyses of CI-AKI incidence based on contrast dose, RIC type, RIC protocol, conditioning organ, hydration, and the definition of CI-AKI. Meta-regression analysis was also performed to evaluate the potential effects of confounders on the renoprotective role of RIC (evaluated by the incidence of CI-AKI). The confounding factors assessed by meta-regression were the history of other disease, baseline SCr, prior contrast administration, duration of RIC, contrast dose, and hydration. Statistical significance of RR and WMD was evaluated with *Z* test, and *P* < 0.05 was considered statistically significant.

Heterogeneity for each outcome analysis was assessed by χ^2^-based Q-test, and the presence of heterogeneity was considered significant if *P* < 0.10 [53]. When the between-study heterogeneity was absent, the fixed effect model (Mantel-Haenszel method) was applied to pool the outcomes from different studies [54]; otherwise, the random effects model (DerSimonian and Laird method) was executed [55]. We also conducted sensitivity analysis to explore the effect of individual study on pooled outcomes and confirm the reliability of results through deleting a single study every time [56]. Begg’s funnel plot and Egger’s linear regression test were performed to investigate the potential publication bias, and *P* < 0.05 indicated statistical significance [57, 58].

In addition, we also conducted a TSA of the incidence of CI-AKI to reduce type I error caused by repetitive significance test of sparse and accumulated data from traditional meta-analyses [59, 60]. TSA was performed with an overall 5% risk of a type I error and a power of 80%, as well as an anticipated 25% RRR. Moreover, the incidence of control arm was estimated after removing high bias risk trials.

For the present analyses, we used Review Manager (version 5.3; Cochrane Collaboration, Oxford, UK), STATA (version 12.0; Stata Corporation, College Station, Texas, USA), and TSA (version 0.9 beta; Copenhagen Trial Unit, Copenhagen, Denmark).

### Quality of evidence

The quality of all evidence for primary and secondary outcomes was estimated using the GRADE instrument with GradePro (version 3.6; http://ims.cochrane.org/revman/gradepro) [61]. The grade assessment of outcomes was categorized as high, moderate, low and very low.

## SUPPLEMENTARY MATERIALS FIGURES AND TABLES







## References

[1] Nash K, Hafeez A, Hou S (2002). Hospital-acquired renal insufficiency. Am J Kidney Dis.

[2] Prasad A, Ortiz-Lopez C, Khan A, Levin D, Kaye DM (2016). Acute kidney injury following peripheral angiography and endovascular therapy: A systematic review of the literature. Catheter Cardiovasc Interv.

[3] Mitchell AM, Jones AE, Tumlin JA, Kline JA (2010). Incidence of contrast-induced nephropathy after contrast-enhanced computed tomography in the outpatient setting. Clin J Am Soc Nephrol.

[4] Chen SL, Zhang J, Yei F, Zhu Z, Liu Z, Lin S, Chu J, Yan J, Zhang R, Kwan TW (2008). Clinical outcomes of contrast-induced nephropathy in patients undergoing percutaneous coronary intervention: a prospective, multicenter, randomized study to analyze the effect of hydration and acetylcysteine. Int J Cardiol.

[5] Turedi S, Erdem E, Karaca Y, Tatli O, Sahin A, Turkmen S, Gunduz A (2016). The high risk of contrast induced nephropathy in patients with suspected pulmonary embolism despite three different prophylaxis: A randomized controlled trial. Acad Emerg Med.

[6] McCullough PA (2008). Contrast-induced acute kidney injury. J Am Coll Cardiol.

[7] Savaj S, Savoj J, Jebraili I, Sezavar SH (2014). Remote ischemic preconditioning for prevention of contrast-induced acute kidney injury in diabetic patients. Iran J Kidney Dis.

[8] Walsh SR, Boyle JR, Tang TY, Sadat U, Cooper DG, Lapsley M, Norden AG, Varty K, Hayes PD, Gaunt ME (2009). Remote ischemic preconditioning for renal and cardiac protection during endovascular aneurysm repair: a randomized controlled trial. J Endovasc Ther.

[9] Thielmann M (2012). Remote ischemic preconditioning in cardiac surgery: caught between clinical relevance and statistical significance?. Basic Res Cardiol.

[10] Meybohm P, Bein B, Brosteanu O, Cremer J, Gruenewald M, Stoppe C, Coburn M, Schaelte G, Böning A, Niemann B, Roesner J, Kletzin F, Strouhal U (2015). A Multicenter Trial of Remote Ischemic Preconditioning for Heart Surgery. N Engl J Med.

[11] Heusch G, Botker HE, Przyklenk K, Redington A, Yellon D (2015). Remote ischemic conditioning. J Am Coll Cardiol.

[12] Saxena P, Newman MA, Shehatha JS, Redington AN, Konstantinov IE (2010). Remote ischemic conditioning: evolution of the concept, mechanisms, and clinical application. J Card Surg.

[13] Selzner N, Boehnert M, Selzner M (2012). Preconditioning, postconditioning, and remote conditioning in solid organ transplantation: basic mechanisms and translational applications. Transplant Rev (Orlando).

[14] Przyklenk K, Bauer B, Ovize M, Kloner RA, Whittaker P (1993). Regional ischemic ‘preconditioning’ protects remote virgin myocardium from subsequent sustained coronary occlusion. Circulation.

[15] Li C, Li YS, Xu M, Wen SH, Yao X, Wu Y, Huang CY, Huang WQ, Liu KX (2013). Limb remote ischemic preconditioning for intestinal and pulmonary protection during elective open infrarenal abdominal aortic aneurysm repair: a randomized controlled trial. Anesthesiology.

[16] Meybohm P, Renner J, Broch O, Caliebe D, Albrecht M, Cremer J, Haake N, Scholz J, Zacharowski K, Bein B (2013). Postoperative neurocognitive dysfunction in patients undergoing cardiac surgery after remote ischemic preconditioning: a double-blind randomized controlled pilot study. PLoS One.

[17] Candilio L, Malik A, Ariti C, Barnard M, Di Salvo C, Lawrence D, Hayward M, Yap J, Roberts N, Sheikh A, Kolvekar S, Hausenloy DJ, Yellon DM (2015). Effect of remote ischaemic preconditioning on clinical outcomes in patients undergoing cardiac bypass surgery: a randomised controlled clinical trial. Heart.

[18] Zarbock A, Schmidt C, Van Aken H, Wempe C, Martens S, Zahn PK, Wolf B, Goebel U, Schwer CI, Rosenberger P, Haeberle H, Gorlich D, Kellum JA (2015). Effect of remote ischemic preconditioning on kidney injury among high-risk patients undergoing cardiac surgery: a randomized clinical trial. Jama.

[19] Huang J, Chen Y, Dong B, Kong W, Zhang J, Xue W, Liu D, Huang Y (2013). Effect of remote ischaemic preconditioning on renal protection in patients undergoing laparoscopic partial nephrectomy: a ‘blinded’ randomised controlled trial. BJU Int.

[20] Pickard JM, Botker HE, Crimi G, Davidson B, Davidson SM, Dutka D, Ferdinandy P, Ganske R, Garcia-Dorado D, Giricz Z, Gourine AV, Heusch G, Kharbanda R (2015). Remote ischemic conditioning: from experimental observation to clinical application: report from the 8th Biennial Hatter Cardiovascular Institute Workshop. Basic Res Cardiol.

[21] Sendeski MM (2011). Pathophysiology of renal tissue damage by iodinated contrast media. Clin Exp Pharmacol Physiol.

[22] Hoole SP, Heck PM, Sharples L, Khan SN, Duehmke R, Densem CG, Clarke SC, Shapiro LM, Schofield PM, O’Sullivan M, Dutka DP (2009). Cardiac Remote Ischemic Preconditioning in Coronary Stenting (CRISP Stent) Study: a prospective, randomized control trial. Circulation.

[23] Tepel M, Zidek W (2004). N-Acetylcysteine in nephrology; contrast nephropathy and beyond. Curr Opin Nephrol Hypertens.

[24] Seeliger E, Sendeski M, Rihal CS, Persson PB (2012). Contrast-induced kidney injury: mechanisms, risk factors, and prevention. Eur Heart J.

[25] Lazaris AM, Maheras AN, Vasdekis SN, Karkaletsis KG, Charalambopoulos A, Kakisis JD, Martikos G, Patapis P, Giamarellos-Bourboulis EJ, Karatzas GM, Liakakos TD (2009). Protective effect of remote ischemic preconditioning in renal ischemia/reperfusion injury, in a model of thoracoabdominal aorta approach. J Surg Res.

[26] Song T, Peng YF, Guo SY, Liu YH, Liul LY (2007). Brief small intestinal ischemia lessens renal ischemia-reperfusion injury in rats. Comp Med.

[27] Koch C, Chaudru S, Lederlin M, Jaquinandi V, Kaladji A, Mahe G (2016). Remote ischemic preconditioning and contrast-induced nephropathy: a systematic review. Ann Vasc Surg.

[28] Zuo B, Wang F, Song Z, Xu M, Wang G (2015). Using remote ischemic conditioning to reduce acute kidney injury in patients undergoing percutaneous coronary intervention: a meta-analysis. Curr Med Res Opin.

[29] Bei WJ, Duan CY, Chen JY, Wang K, Liu YH, Liu Y, Tan N (2016). Remote Ischemic Conditioning for Preventing Contrast-Induced Acute Kidney Injury in Patients Undergoing Percutaneous Coronary Interventions/Coronary Angiography: A Meta-Analysis of Randomized Controlled Trials. J Cardiovasc Pharmacol Ther.

[30] Yamanaka T, Kawai Y, Miyoshi T, Mima T, Takagaki K, Tsukuda S, Kazatani Y, Nakamura K, Ito H (2015). Remote ischemic preconditioning reduces contrast-induced acute kidney injury in patients with ST-elevation myocardial infarction: A randomized controlled trial. Int J Cardiol.

[31] Menting TP, Sterenborg TB, de Waal Y, Donders R, Wever KE, Lemson MS, van der Vliet JA, Wetzels JF, SchultzeKool LJ, Warle MC (2015). Remote Ischemic Preconditioning To Reduce Contrast-Induced Nephropathy: A Randomized Controlled Trial. Eur J Vasc Endovasc Surg.

[32] Healy DA, Feeley I, Keogh CJ, Scanlon TG, Hodnett PA, Stack AG, Moloney MC, Whittaker P, Walsh SR (2015). Remote ischemic conditioning and renal function after contrast-enhanced CT scan: A randomized trial. Clin Invest Med.

[33] Xu X, Zhou Y, Luo S, Zhang W, Zhao Y, Yu M, Ma Q, Gao F, Shen H, Zhang J (2014). Effect of remote ischemic preconditioning in the elderly patients with coronary artery disease with diabetes mellitus undergoing elective drug-eluting stent implantation. Angiology.

[34] Lavi S, D’Alfonso S, Diamantouros P, Camuglia A, Garg P, Teefy P, Jablonsky G, Sridhar K, Lavi R (2014). Remote Ischemic Postconditioning During Percutaneous Coronary Interventions Remote Ischemic Postconditioning-Percutaneous Coronary Intervention Randomized Trial. Circ Cardiovasc Interv.

[35] Crimi G, Ferlini M, Gallo F, Sormani MP, Raineri C, Bramucci E, De Ferrari GM, Pica S, Marinoni B, Repetto A, Raisaro A, Leonardi S, Rubartelli P (2014). Remote ischemic postconditioning as a strategy to reduce acute kidney injury during primary PCI: A post-hoc analysis of a randomized trial. Int J Cardiol.

[36] Luo SJ, Zhou YJ, Shi DM, Ge HL, Wang JL, Liu RF (2013). Remote ischemic preconditioning reduces myocardial injury in patients undergoing coronary stent implantation. Can J Cardiol.

[37] Igarashi G, Iino K, Watanabe H, Ito H (2013). Remote ischemic pre-conditioning alleviates contrast-induced acute kidney injury in patients with moderate chronic kidney disease. Circ J.

[38] Deftereos S, Giannopoulos G, Tzalamouras V, Raisakis K, Kossyvakis C, Kaoukis A, Panagopoulou V, Karageorgiou S, Avramides D, Toutouzas K, Hahalis G, Pyrgakis V, Manolis AS (2013). Renoprotective effect of remote ischemic post-conditioning by intermittent balloon inflations in patients undergoing percutaneous coronary intervention. J Am Coll Cardiol.

[39] Er F, Nia AM, Dopp H, Hellmich M, Dahlem KM, Caglayan E, Kubacki T, Benzing T, Erdmann E, Burst V, Gassanov N (2012). Ischemic preconditioning for prevention of contrast medium-induced nephropathy: randomized pilot RenPro Trial (Renal Protection Trial). Circulation.

[40] Gholoobi A, Sajjadi SM, Shabestari MM, Eshraghi A, Shamloo AS (2015). The Impact of Remote Ischemic Pre-Conditioning on Contrast-Induced Nephropathy in Patients Undergoing Coronary Angiography and Angioplasty: A Double-Blind Randomized Clinical Trial. Electron Physician.

[41] Balbir Singh G, Ann SH, Park J, Chung HC, Lee JS, Kim ES, Choi JI, Lee J, Kim SJ, Shin ES (2016). Remote Ischemic Preconditioning for the Prevention of Contrast-Induced Acute Kidney Injury in Diabetics Receiving Elective Percutaneous Coronary Intervention. PLoS One.

[42] Kahlert P, Hildebrandt HA, Patsalis PC, Al-Rashid F, Janosi RA, Nensa F, Schlosser TW, Schlamann M, Wendt D, Thielmann M, Kottenberg E, Frey U, Neuhauser M (2017). No protection of heart, kidneys and brain by remote ischemic preconditioning before transfemoral transcatheter aortic valve implantation: Interim-analysis of a randomized single-blinded, placebo-controlled, single-center trial. Int J Cardiol.

[43] Higgins JP, Altman DG, Gotzsche PC, Juni P, Moher D, Oxman AD, Savovic J, Schulz KF, Weeks L, Sterne JA, Cochrane Bias Methods G, Cochrane Statistical Methods G (2011). The Cochrane Collaboration’s tool for assessing risk of bias in randomised trials. BMJ.

[44] Evans RG, Ince C, Joles JA, Smith DW, May CN, O’Connor PM, Gardiner BS (2013). Haemodynamic influences on kidney oxygenation: clinical implications of integrative physiology. Clin Exp Pharmacol Physiol.

[45] Marenzi G, Assanelli E, Campodonico J, Lauri G, Marana I, De Metrio M, Moltrasio M, Grazi M, Rubino M, Veglia F, Fabbiocchi F, Bartorelli AL (2009). Contrast volume during primary percutaneous coronary intervention and subsequent contrast-induced nephropathy and mortality. Ann Intern Med.

[46] Ovize M, Baxter GF, Di Lisa F, Ferdinandy P, Garcia-Dorado D, Hausenloy DJ, Heusch G, Vinten-Johansen J, Yellon DM, Schulz R, Working Group of Cellular Biology of Heart of European Society of C (2010). Postconditioning and protection from reperfusion injury: where do we stand? Position paper from the Working Group of Cellular Biology of the Heart of the European Society of Cardiology. Cardiovasc Res.

[47] Dong S, Cao Y, Li H, Tian J, Yi C, Sang W (2015). Impact of ischemic preconditioning on ischemia-reperfusion injury of the rat sciatic nerve. Int J Clin Exp Med.

[48] Lu Y, Dong CS, Yu JM, Li H (2012). Morphine reduces the threshold of remote ischemic preconditioning against myocardial ischemia and reperfusion injury in rats: the role of opioid receptors. J Cardiothorac Vasc Anesth.

[49] Xin P, Zhu W, Li J, Ma S, Wang L, Liu M, Li J, Wei M, Redington AN (2010). Combined local ischemic postconditioning and remote perconditioning recapitulate cardioprotective effects of local ischemic preconditioning. Am J Physiol Heart Circ Physiol.

[50] Loukogeorgakis SP, Williams R, Panagiotidou AT, Kolvekar SK, Donald A, Cole TJ, Yellon DM, Deanfield JE, MacAllister RJ (2007). Transient limb ischemia induces remote preconditioning and remote postconditioning in humans by a K(ATP)-channel dependent mechanism. Circulation.

[51] Waikar SS, Bonventre JV (2009). Creatinine kinetics and the definition of acute kidney injury. J Am Soc Nephrol.

[52] Moher D, Liberati A, Tetzlaff J, Altman DG, Group P (2009). Preferred reporting items for systematic reviews and meta-analyses: the PRISMA statement. BMJ.

[53] Higgins JP, Thompson SG, Deeks JJ, Altman DG (2003). Measuring inconsistency in meta-analyses. BMJ.

[54] Mantel N, Haenszel W (1959). Statistical aspects of the analysis of data from retrospective studies of disease. J Natl Cancer Inst.

[55] DerSimonian R, Laird N (1986). Meta-analysis in clinical trials. Control Clin Trials.

[56] Thakkinstian A, McElduff P, D’Este C, Duffy D, Attia J (2005). A method for meta-analysis of molecular association studies. Stat Med.

[57] Begg CB, Mazumdar M (1994). Operating characteristics of a rank correlation test for publication bias. Biometrics.

[58] Egger M, Davey Smith G, Schneider M, Minder C (1997). Bias in meta-analysis detected by a simple, graphical test. BMJ.

[59] Brok J, Thorlund K, Wetterslev J, Gluud C (2009). Apparently conclusive meta-analyses may be inconclusive—Trial sequential analysis adjustment of random error risk due to repetitive testing of accumulating data in apparently conclusive neonatal meta-analyses. Int J Epidemiol.

[60] Holst LB, Petersen MW, Haase N, Perner A, Wetterslev J (2015). Restrictive versus liberal transfusion strategy for red blood cell transfusion: systematic review of randomised trials with meta-analysis and trial sequential analysis. BMJ.

[61] Guyatt GH, Oxman AD, Vist GE, Kunz R, Falck-Ytter Y, Alonso-Coello P, Schunemann HJ, Group GW (2008). GRADE: an emerging consensus on rating quality of evidence and strength of recommendations. BMJ.

