# Supplementary material for: Remote ischemic conditioning for the prevention of contrast-induced acute kidney injury in patients undergoing intravascular contrast administration: a meta-analysis and trial sequential analysis of 16 randomized controlled trials
Source: Oncotarget. 2017 May 23;8(45):79323–36. doi: 10.18632/oncotarget.18106 (PMC5668044; doi:10.18632/oncotarget.18106)
Supplement: Supplementary file 1 [file oncotarget-08-79323-s001.pdf]

Remote ischemic conditioning for the prevention of contrast-induced acute kidney injury in patients undergoing intravascular contrast administration: a meta-analysis and trial sequential analysis of 16 randomized controlled trials

Supplementary Materials

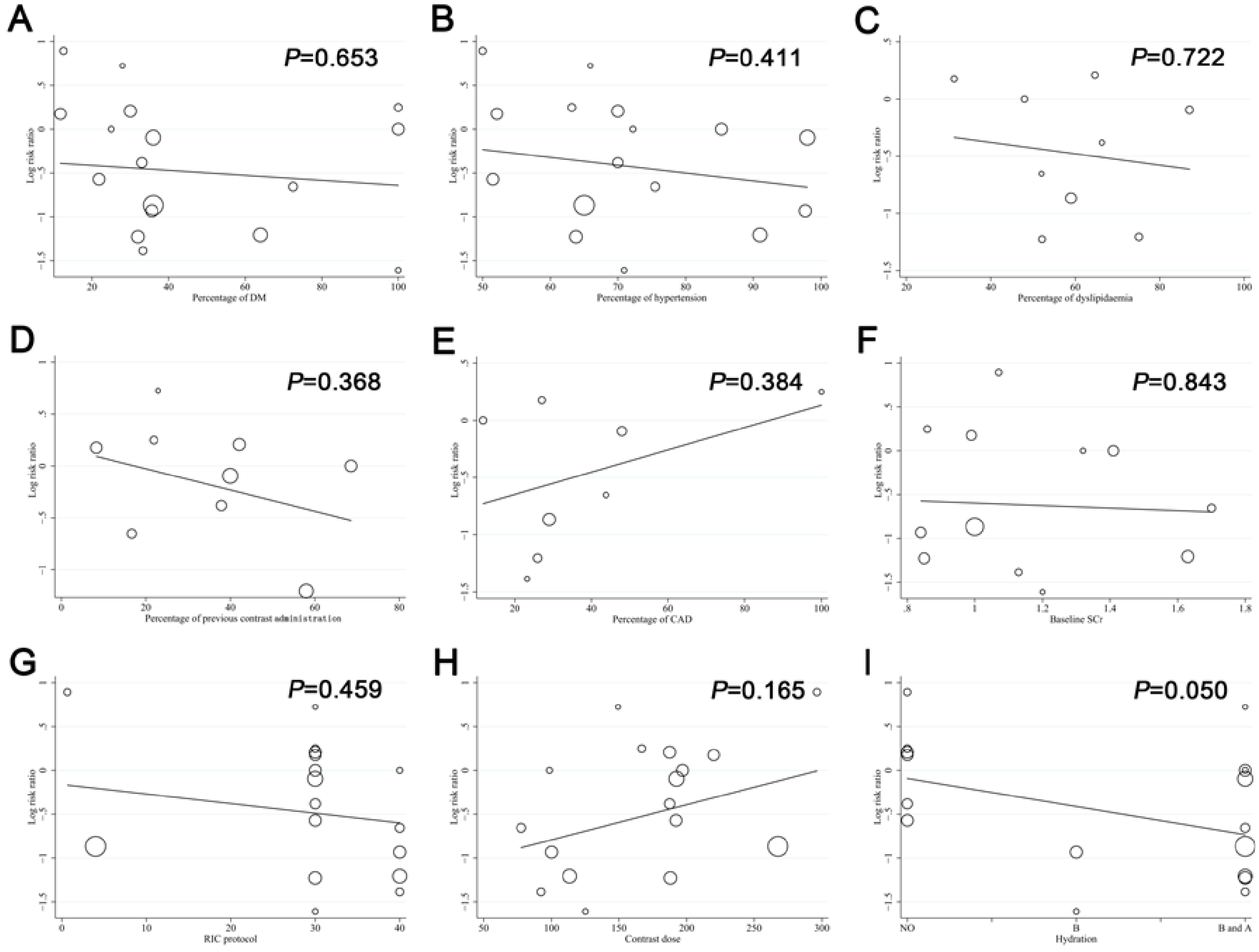

Supplementary Figure 1: Meta-regression results of reduction of contrast-induced acute kidney injury (CI-AKI) by remote ischemic conditioning (RIC). (**A**) percentage of DM; (**B**) percentage of hypertension; (**C**) percentage of dyslipidaemia; (**D**) percentage of previous contrast administration; (**E**) percentage of CAD; (**F**) baseline SCr; (**G**) duration of RIC; (**H**) mean contrast dose; (**I**) hydration. DM: diabetes mellitus; SCr: serum creatinine; CAD: coronary artery disease.

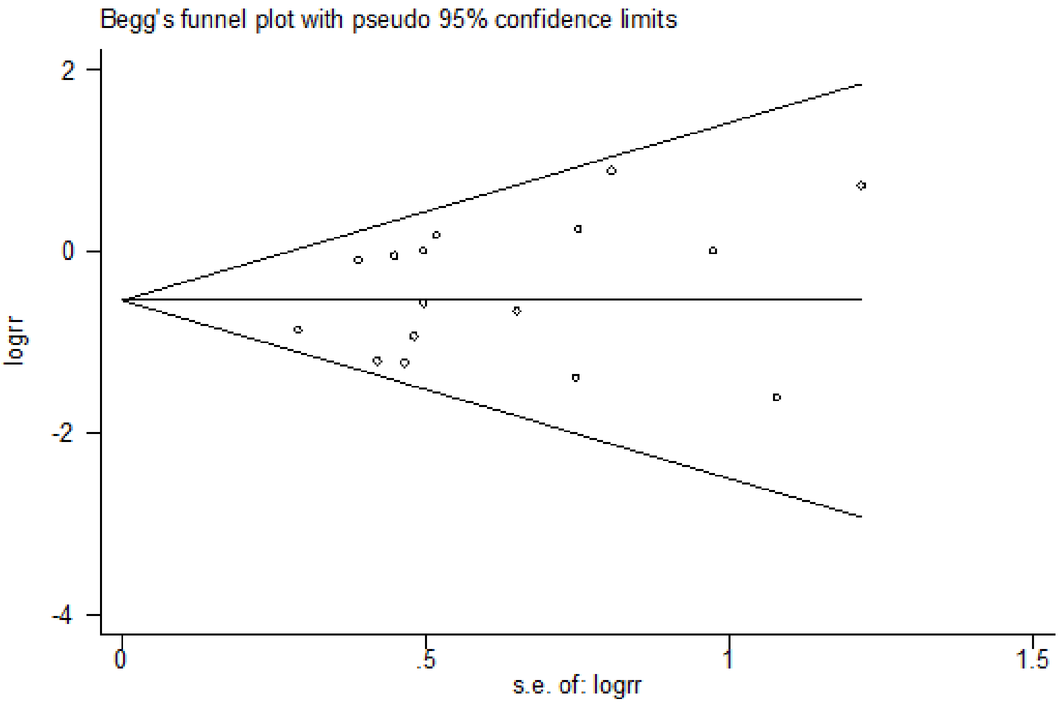

Supplementary Figure. S2. Begg's funnel plot for publication bias test of CI-AKI incidence. Description: the funnel plot was symmetrical, suggesting the absence of publication bias.

For Supplementary Tables see in Supplementary Files.
