# Supplementary material for: Remote ischemic conditioning for the prevention of contrast-induced acute kidney injury in patients undergoing intravascular contrast administration: a meta-analysis and trial sequential analysis of 16 randomized controlled trials
Source: Oncotarget. 2017 May 23;8(45):79323–36. doi: 10.18632/oncotarget.18106 (PMC5668044; doi:10.18632/oncotarget.18106)
Supplement: Supplementary file 2 [file oncotarget-08-79323-s002.docx]

**SUPPLEMENTARY TABLES AND FIGURES**

**Supplementary Table S1. Characteristics of included trials**

| **Author** | **Year** | **DM**  **(%)** | **Hypertension (%)** | **Dyslipidaemia (%)** | **Previous contrast administration (%)** | **CAD**  **(%)** | **Baseline SCr (mg/dl)** |
| --- | --- | --- | --- | --- | --- | --- | --- |
|  |  |  |  |  |  |  |  |
| Kahlert | 2017 | 36 | 98 | 87 | 40 | 48 | - |
| Balbir | 2016 | 100 | 85.3 | 48 | 68.6 | 11.8 | 1.41 ± 0.48 |
| Yamanaka | 2015 | 32 | 63.8 | 52.1 | - | - | 0.85 |
| Menting | 2015 | 25 | 72.2 | - | - | - | 1.32 |
| Healy | 2015 | 35.6 | 97.7 | - | - | - | 0.84±0.19 |
| Gholoobi | 2015 | 72.5 | 75.5 | 52 | 16.7 | 43.8 | 1.7 |
| Xu | 2014 | 100 | 63.2 | - | 21.9 | 100 | 0.86 |
| Savaj | 2014 | 100 | 70.9 | - | - | - | 1.2 |
| Lavi 1 | 2014 | 33 | 70 | 66.3 | 37.9 | - | - |
| Lavi 2 | 2014 | 30 | 70 | 64.6 | 42.1 | - | - |
| Crimi | 2014 | 11.7 | 52.1 | 31.3 | 8.3 | 27.1 | 0.99 |
| Luo | 2013 | 28 | 65.9 | - | 22.9 | - | - |
| Igarashi | 2013 | 33.3 | - | - | - | 23.3 | 1.13±0.24 |
| Deftereos | 2013 | 36 | 65 | 59 | - | 29 | 1±0.3 |
| Er | 2012 | 64 | 91 | 75 | 58 | 26 | 1.63 |
| Walsh | 2009 | 12.5 | 50 | - | - | - | 1.07 |
| Hoole | 2009 | 21.8 | 51.5 | - | - | - | - |

DM: diabetes mellitus; CAD: coronary artery disease; SCr: serum creatinine.

**Supplementary Table S2. Statistical analyses of publication bias**

| **Category** | **CI-AKI** | **SCr-24h** | **SCr-48h** | **Mortaity** | **MACEs** |
| --- | --- | --- | --- | --- | --- |
| Begg's Test | 0.444 | 1 | 1 | 0.902 | 0.881 |
| Egger's test | 0.279 | 0.723 | 0.503 | 0.155 | 0.796 |

Publication bias was determined by *P* value, and *P*<0.05 was considered statistically significant. CI-AKI: contrast-induced acute kidney injury; SCr: serum creatinine; MACEs: major adverse cardiovascular events.

**Supplementary Table S3: GRADE assessment of all outcomes**

| **Quality assessment** | | | | | | | **No of patients** | | **Effect** | | **Quality** | **Importance** |
| --- | --- | --- | --- | --- | --- | --- | --- | --- | --- | --- | --- | --- |
|  |  |  |  |  |  |  |  |  |  |  |  |  |
| **No of studies** | **Design** | **Risk of bias** | **Inconsistency** | **Indirectness** | **Imprecision** | **Other considerations** | **Other** | **Control** | **Relative (95% CI)** | **Absolute** |  |  |
| **CI-AKI** | | | | | | | | | | | | |
| 16 | randomised trials | serious^1^ | No serious inconsistency | No serious indirectness | No serious imprecision | none | 92/1093  (8.4%) | 158/1082  (14.6%) | RR 0.58 (0.46 to 0.74) | 61 fewer per 1000 (from 38 fewer to 79 fewer) |  MODERATE | CRITICAL |
|  |  |  |  |  |  |  |  | 12.5% |  | 53 fewer per 1000 (from 32 fewer to 67 fewer) |  |  |
| **SCr-24h** | | | | | | | | | | | | |
| 5 | randomised trials | serious^2^ | No serious inconsistency | No serious indirectness | No serious imprecision | none | 403 | 408 | - | MD 0 higher (0.03 lower to 0.04 higher) |  MODERATE | IMPORTANT |
| **SCr-48h** | | | | | | | | | | | | |
| 6 | randomised trials | no serious risk of bias | No serious inconsistency | No serious indirectness | serious^3^ | none | 221 | 226 | - | MD 0.1 lower (0.18 to 0.02 lower) |  MODERATE | IMPORTANT |
| **mortality** | | | | | | | | | | | | |
| 8 | randomised trials | no serious risk of bias | No serious inconsistency | No serious indirectness | serious^3^ | none | 5/466  (1.1%) | 13/463  (2.8%) | RR 0.51 (0.22 to 1.17) | 14 fewer per 1000 (from 22 fewer to 5 more) |  MODERATE | IMPORTANT |
|  |  |  |  |  |  |  |  | 3% |  | 15 fewer per 1000 (from 23 fewer to 5 more) |  |  |
| **MACEs** | | | | | | | | | | | | |
| 7 | randomised trials | no serious risk of bias | No serious inconsistency | serious^4^ | No serious imprecision | none | 47/430  (10.9%) | 81/427  (19%) | RR 0.58 (0.42 to 0.80) | 80 fewer per 1000 (from 38 fewer to 110 fewer) |  MODERATE | IMPORTANT |
|  |  |  |  |  |  |  |  | 14.9% |  | 63 fewer per 1000 (from 30 fewer to 86 fewer) |  |  |

^1^ Two trials were involved in relatively high risk of bias.
^2^ One trials were involved in relatively high risk of bias.
^3^ The width of corresponding 95% confidence interval was large
^4^ The outcomes of MACE were not strictly defined in some trials and might contain some all cause adverse events.

CI-AKI, contrast-induced acute kidney injury; SCr, serum creatinine; MACEs, major adverse cardiovascular events.
